# ProCAID: a phase I clinical trial to combine the AKT inhibitor AZD5363 with docetaxel and prednisolone chemotherapy for metastatic castration resistant prostate cancer

**DOI:** 10.1007/s10637-017-0433-4

**Published:** 2017-02-01

**Authors:** Simon J. Crabb, Alison J. Birtle, Karen Martin, Nichola Downs, Ian Ratcliffe, Tom Maishman, Mary Ellis, Gareth Griffiths, Stuart Thompson, Lidia Ksiazek, Vincent Khoo, Robert J. Jones

**Affiliations:** 1 Southampton Experimental Cancer Medicine Centre, University of Southampton, Southampton, UK; 2 Lancashire Teaching Hospitals NHS Foundation Trust, Preston, UK; 3 Southampton Clinical Trials Unit, University of Southampton, Southampton, UK; 4 Patient and Public Involvement Representative, Southampton, UK; 5 The Royal Marsden NHS Foundation Trust, London, UK; 6 The Beatson West of Scotland Cancer Centre, Glasgow, UK

**Keywords:** Castration resistant prostate cancer, Metastatic, Docetaxel, AZD5363, Phase I, AKT

## Abstract

*Background* Docetaxel and prednisolone chemotherapy (DP) extends survival in metastatic castration resistant prostate cancer (mCRPC). However, emergent clinical resistance is almost inevitable. AKT pathway activation is highly prevalent in mCRPC contributing to disease progression and DP resistance. AZD5363 is a potent oral pan-AKT inhibitor with pre-clinical data indicating activity in mCRPC and synergy with docetaxel. *Methods* This phase I trial was to determine an AZD5363 recommended phase II dose (RP2D) for combination with DP. Eligibility criteria included chemotherapy naive mCRPC, PSA or radiographic disease progression and ECOG performance status 0 or 1. Treatment comprised DP (75 mg/m^2^, IV, day 1 and 5 mg BID, PO, day 1–21 respectively for ten cycles) and AZD5363 to disease progression for all patients. We utilised a 3 + 3 dose escalation design to determine a maximum tolerated dose according to defined dose limiting toxicity criteria assessed using CTCAE version 4.03. Planned AZD5363 dose levels were 320 mg (DL1), 400 mg (DL2) and 480 mg (DL3), BID, PO, 4 days on/3 days off, from day 2 of each cycle. *Results* 10 patients were treated. Dose limiting toxicities affected 2 patients (grade 3 rash ≥5 days; grade 3 diarrhoea) in DL2. The commonest grade 3 or 4, AZD5363 related, symptomatic adverse events were rash and diarrhoea. Hyperglycaemia affected all patients but was self-limiting. PSA reduction to <50% at 12 weeks occurred in 7 patients. *Conclusions* The RP2D for AZD5363 is 320 mg BID, 4 days on/3 days off, in combination with full dose DP for mCRPC.

**Electronic supplementary material:**

The online version of this article (doi:10.1007/s10637-017-0433-4) contains supplementary material, which is available to authorized users.

## Introduction

There are approximately 47,000 new cases and 11,000 deaths from prostate cancer in the UK per year [1]. Docetaxel and prednisolone chemotherapy (DP) extends survival and maintains quality of life in metastatic castration resistant prostate cancer (mCRPC) [2, 3]. However, despite the advent of chemotherapy utilisation earlier in the treatment pathway for hormone sensitive disease, and the introduction of multiple other life extending therapeutic options, advanced prostate cancer remains incurable [4–12]. From the point of castrate resistance, median survival is in the range of 2–3 years [4–6]. One contributory factor in these outcomes is the common phenomenon of emergent therapeutic resistance either during, or shortly following, the administration of DP for mCRPC. For example, in a 755 patient phase III study evaluating subsequent chemotherapy, three quarters of patients had progressed during or within 3 months of completing DP [8]. A pressing unmet need, therefore, remains to develop therapeutic strategies to address clinical resistance to DP and improve on current outcomes.

Serine/threonine protein kinase AKT (protein kinase B) pathway activation is highly prevalent in prostate cancer. In addition to the loss of PTEN function through deletion, inactivating mutation, or reduced protein expression, the frequency of pathway alteration rises substantially when inactivating PHLPP and INPP4B alterations or activating PI3K component (PIK3R1, PIK3R3, PIK3CA) and AKT isoform mutations are also included as potential causes of pathway activation [13]. Data indicate that the rate of a potentially activating pathway alteration rises to approaching 100% if assessed within metastatic samples. Furthermore, in pre-clinical models AKT pathway activation has been shown to contribute to disease progression and therapeutic resistance to DP [13–15].

AZD5363 is a potent oral pan-AKT inhibitor with activity against AKT 1, 2 and 3 (IC_50_ < 10 nM). It also inhibits protein kinase A (PKA) and the Rho associated protein kinases (ROCK1 and 2). AZD5363 inhibits phosphorylation of AKT substrates GSK3β and PRAS40 and the downstream biomarker S6 in cell lines, including LNCaP prostate cancer cells and rodent xenografts including from PC3 prostate cancer cells. Pre-clinical data support both single agent activity in mCRPC and synergy with docetaxel [14, 16, 17]. In first in human studies as a single agent, diarrhoea, hyperglycaemia, nausea, and rash were the most common adverse events (AE) [18, 19]. Exploratory data from these single agent trials is consistent with relevant signalling inhibition based on pre- and post-treatment tumour biopsies (increase in phospho-AKT, reduction in phospho-GSK3β and phospho-PRAS40) and clinical activity in patients with PIK3CA-mutant breast cancer and in patients with tumour AKT1 (E17K) mutations [18, 19].

The ProCAID phase I clinical trial was undertaken to establish a DP/AZD5363 combination to allow for subsequent development in mCRPC and potentially also earlier phases of the disease pathway.

## Patients and methods

### Patients

Eligible patients were 18 years or older with histologically or cytologically proven mCRPC and an Eastern Cooperative Oncology Group performance status of 0 or 1. All patients had disease progression based on PSA and/or radiographic criteria defined by the Response Evaluation Criteria in Solid Tumors (RECIST, version 1.1) and the Prostate Cancer Working Group 2 [20, 21]. Radiologically measurable and/or evaluable disease was acceptable. Patients were required to have a serum testosterone <1.7 nmol/L and ongoing LHRH analogue or antagonist therapy was permitted to maintain a castrate state. Other therapies for prostate cancer, other than ongoing bisphosphonates or denosumab, were discontinued ≥4 weeks prior to commencing study treatment and anti-androgen withdrawal response was excluded where relevant. Haematological parameter requirements were: haemoglobin ≥9 g/dL, platelets ≥100 × 10^9^/L, neutrophils ≥1.5 × 10^9^/L, bilirubin ≤ the institutional upper limit of normal (ULN), alanine (ALT) and aspartate (AST) aminotransferase ≤1.5 x ULN and sodium and potassium within the normal range for the treating institution. Patients were excluded if they had received previous treatment with cytotoxic chemotherapy but were permitted the prior use of second generation hormonal therapies e.g., abiraterone or enzalutamide. Other exclusion criteria included prior malignancy with an estimated ≥30% chance of relapse within 2 years, previously identified brain metastases, or spinal cord compression unless treated with full functional recovery, prior radiotherapy to >30% of bone marrow, another investigational agent within 30 days of study medication, type I or II diabetes mellitus requiring either insulin or oral hypoglycaemics for routine management, gastrointestinal conditions that might affect drug absorption, significant cardiac disease within the last 6 months, a left ventricular ejection fraction < the institutional lower limit of normal, a QTc interval of >480 msec, or recent exposure to potent inhibitors or inducers of CYP3A4 or substrates of CYP3A4 and CYP2D6. The complete eligibility criteria are listed in the Supplementary Appendix.

### Treatment

**Fig. 1:**
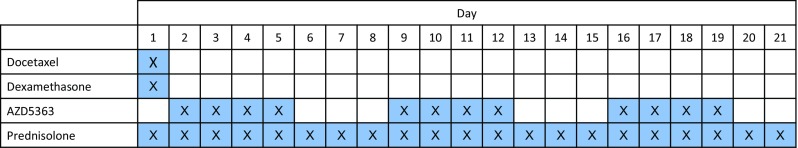
Schematic representation of the days of dosing (*blue crossed boxes*) for each of the indicated drugs at the following doses: docetaxel, 75 mg/m^2^ by one hour intravenous infusion on day 1; dexamethasone 8 mg orally, at 12, 3 and 1 h prior to each docetaxel infusion; AZD5363, dosed according to dose level cohort, BID, orally, taken 4 days on/3 days off, continuously from cycle 1 day 2; prednisolone, 5 mg BID orally days 1–21

The treatment cycle is summarised in Fig. 1 and comprised DP for up to 10 cycles of 21 days. All patients received docetaxel 75 mg/m^2^ by one hour intravenous infusion on day 1 and prednisolone 5 mg BID, orally, on days 1 to 21 of each cycle. In addition, patients received AZD5363 which was continued until either disease progression, the commencement of new anti-prostate cancer systemic therapy or unacceptable toxicity. Planned AZD5363 dose levels were 320 mg (dose level 1), 400 mg (dose level 2) and 480 mg (dose level 3), BID, orally, given according to a 4 days on and 3 days off schedule which commenced from cycle 1, day 2. Dexamethasone pre-medication was recommended at 8 mg, orally, at 12, 3 and 1 h prior to each docetaxel infusion and anti-emetics were given according to local institutional protocols. Body surface area calculations and re-calculations due to changes in weight and docetaxel dose banding (up to +/− 5%) were permitted in accordance with local institutional practices.

### Study design

We utilised a conventional 3 + 3 dose escalation phase I clinical trial design. An evaluable patient was defined as one that completed cycle 1 and received at least 80% of the total cumulative doses of AZD5363 and prednisolone, and the full dose of docetaxel, with no more than a 14-day delay in starting cycle 2, or who had experienced a dose limiting toxicity (DLT). AE data was recorded for all patients who commenced study treatment.

The National Cancer Institute Common Toxicity Criteria for Adverse Events version 4.03 (CTCAEv4.03, http://evs.nci.nih.gov/ftp1/CTCAE/CTCAE_4.03_2010-06-14_QuickReference_5x7.pdf) was used to characterise AEs. DLTs were predefined and based on recorded AEs as: a greater than 14 day delay in administration of docetaxel for cycle 2 due to drug toxicity; grade 4 neutropenia ≥7 days duration; grade 3 or 4 neutropenia associated with a temperature ≥ 38.5 °C; grade 3 or 4 neutropenia associated with bacteriologically proven sepsis; any grade 4 thrombocytopenia; grade 3 thrombocytopenia associated with non-traumatic bleeding (except where this could be explained by therapeutic anticoagulation); ≥ grade 3 hyperglycaemia for more than 1 week despite optimal intervention; grade 4 hyperglycaemia; AST or ALT >10 x ULN where AZD5363 was considered the most likely cause; AST or ALT >8 x ULN when combined with a doubling of bilirubin from baseline and where AZD5363 was considered the most likely cause; QTc (Fridericia’s or Bazett’s correction) interval > 500 msec or QTc increase >60 msec from baseline on two ECGs at least 30 min apart that could not be attributed to another cause; symptomatic congestive cardiac failure (New York Heart Association class III/IV) and a drop in LVEF that could not be attributed to another cause; a decrease in LVEF of ≥20% to a level below the institutional lower limit of normal range; clinically significant rash that despite optimal treatment remained grade ≥ 3 for 5 days or longer and that could not be attributed to another cause; grade ≥ 3 nausea, vomiting or diarrhoea, despite optimal anti-emetic or anti-diarrhoeal therapy and which could not be attributed to another cause; any other grade ≥ 3 toxicity which in the opinion of the investigator was clinically significant and related to AZD5363. Designation of a DLT excluded isolated laboratory changes of any grade (except as specified above) without clinical sequelae or clinical significance.

Intra-patient dose escalation was not permitted. In addition to protocol defined DLTs, the protocol allowed for the Safety Review Committee to consider other toxicities including those emerging during subsequent treatment cycles in prior patients in making recommendations. Dose escalation was undertaken if none of 3, or 1 of 6, evaluable patients experienced a DLT at the current dose level. If 2 or more patients experienced a DLT then the preceding dose level was established as the maximum tolerated dose (MTD). The recommended phase II dose (RP2D) was the MTD or dose level three if this was tolerated.

The primary endpoint was the determination of an RP2D for AZD5363, using a four days on/three days off administration schedule, in combination with full dose DP. Secondary endpoints included safety and tolerability profiles using CTCAEv4.03 and AZD5363 pharmacokinetics when combined with DP. Results were summarised descriptively.

Trial conduct was consistent with Good Clinical Practice guidelines and the Declaration of Helsinki and following national ethics and regulatory approvals. Informed consent was obtained from all individual participants included in the study. The trial was coordinated by the Cancer Research UK Southampton Clinical Trials Unit and sponsored by University Hospital Southampton NHS Foundation Trust.

## Results

### Patients

Ten patients with a median age of 67.5 (range 56–72) were recruited from two UK centres with 4 patients in dose level 1, and 6 in dose level 2. Nine patients (90%) had bony metastases and 5 (50%) had visceral metastases. One patient in dose level 1 was considered to be non-evaluable for DLT assessment due to a compliance error, resulting from misunderstanding rather than treatment related AEs, in cycle 1 resulting in only 54.2% of the cumulative AZD5363 dose being taken. This patient subsequently tolerated treatment at full dose from cycle 2 onwards. Patient characteristics are shown in Table 1.

**Table 1:** Patient characteristics

Dose level	320 mg (*n* = 4)	400 mg (*n* = 6)	All patients (*n* = 10)
Age			
Median, years (range)	66.5 (56–68)	68 (62–72)	67.5 (56–72)
≥ 65 years, n (%)	3 (75%)	4 (67%)	7 (70%)
ECOG performance status, n (%)			
0	3 (75%)	4 (67%)	7 (70%)
1	1 (25%)	2 (33%)	3 (30%)
Gleason score at diagnosis, n (%)*			
≤ 6	0	0	0
7	2 (50%)	2 (33%)	4 (40%)
8–10	2 (50%)	3 (50%)	5 (50%)
Metastatic sites, n (%)			
Bone only	2 (50%)	3 (50%)	5 (50%)
Visceral only	1 (25%)	0	1 (10%)
Visceral and bone	1 (25%)	3 (50%)	4 (40%)
Prior abiraterone or enzalutamide			
n (%)	0	3 (50%)	3 (30%)
PSA, μg/L			
Median, (range)	175 (76–320)	115 (0.7–620)	115 (0.7–620)
Haemoglobin, g/L			
Median, (range)	136.5 (132–160)	133 (105–147)	134.5 (105–160)
Alkaline Phosphatase (U/L)			
Median (range)	118 (74–524)	151.5 (76–720)	135 4–720)

Abbreviations *ECOG* Eastern Cooperative Oncology Group, *PSA* prostate specific antigen

*Gleason score was not recorded for one patient (who had a confirmed diagnosis of cancer from a prostate biopsy)

### Dose escalation and safety

A median of 8.5 cycles (range 3–10) of DP and 4 cycles (range 1–13) of AZD5363 was administered. The actual number of cycles administered for each patient is shown in Table 2. In dose level 1, all 3 evaluable patients received an equal or greater number of cycles of AZD5363 compared to DP. In dose level 2 however, this was the case for only 2 of 6 patients. The 4 other patients in dose level 2 received only 1 or 2 cycles of AZD5363 whilst receiving between 3 and 10 cycles of docetaxel. No DLTs were seen in dose level 1. Two patients in dose level 2 experienced DLTs. These were due to grade 3 rash for ≥5 days and grade 3 diarrhoea despite optimal anti-diarrhoeal therapy. The severity and number of AEs overall and by dose level is shown in Table 3. Five patients in cycle 1, and 8 (80%) across all cycles had at least one grade 3 or 4 AE.

**Table 2:** Number of cycles of DP and AZD5363 receved for each patient

Patient	Dose level	Number of cycles of DP administered	Number of cycles of AZD5363 administered
051001	1	10	3
051002	1	7	7
051003	1	10	10
051004	1	10	13
051005	2	10	2
051006	2	6	1
028007	2	10	13
051008	2	5	5
051009	2	3	1
051010	2	4	2

**Table 3:** Adverse events (by CTCAE version 4.03) summary

Dose level	320 mg (*n* = 4)	400 mg (*n* = 6)	All patients (*n* = 10)
Cycle 1			
Number of AEs per patient			
Median	7.5	11.5	9.5
Range	3–14	4–33	3–33
Worst CTCAE grade experienced, n (%)			
1 – Mild	1 (25%)	2 (33%)	3 (30%)
2 – Moderate	2 (50%)	0	2 (20%)
3 – Severe	1 (25%)	3 (50%)	4 (40%)
4 – Life threatening	0	1 (17%)	1 (10%)
5 – Death	0	0	0
At least one grade ≥ 3 event	1 (25%)	4 (67%)	5 (50%)
All cycles			
Number of AEs per patient			
Median	23	23.5	23.5
Range	17–26	22–76	17–76
Worst CTCAE grade experienced, n (%)			
1 – Mild	0	0	0
2 – Moderate	1 (25%)	1 (17%)	2 (20%)
3 – Severe	3 (75%)	4 (67%)	7 (70%)
4 – Life threatening	0	1 (17%)	1 (10%)
5 – Death	0	0	0
At least one grade ≥ 3 event	3 (75%)	5 (83%)	8 (80%)

Abbreviations: AE, adverse event; CTCAE, Common Toxicity Criteria for Adverse Events

Rates of specific AEs, occurring in at least one patient, are shown for cycle 1 (Table 4) and across all cycles (Table 5). Across all cycles, the most common grade 3 or 4 AEs considered by the investigator to be related to AZD5363 were maculopapular rash and diarrhoea (3 patients and 2 patients respectively). In most cases of rash and diarrhoea, utilisation of systemic anti-histamines and loperamide respectively allowed for successful management and to allow ongoing administration of study medication. Febrile neutropenia occurred in 2 (20%) of patients across all treatment cycles. There were no treatment related deaths.

**Table 4:** Adverse events (CTCAE version 4.03), regardless of causality, occurring during cycle 1 of treatment*

Dose level	320 mg (*n* = 4)		400 mg (*n* = 6)		All patients (*n* = 10)	
	Grade ≥ 1	Grade ≥ 3	Grade ≥ 1	Grade ≥ 3	Grade ≥ 1	Grade ≥ 3
Diarrhoea	3 (75%)	0	5 (83%)	2 (33%)	8 (80%)	2 (20%)
Rash	2 (50%)	0	4 (67%)	3 (50%)	6 (60%)	3 (30%)
Neutropenia	0	0	2 (33%)	2 (33%)	2 (20%)	2 (20%)
Fever	0	0	2 (33%)	1 (17%)	2 (20%)	1 (10%)
Febrile neutropenia	0	0	1 (17%)	1 (17%)	1 (10%)	1 (10%)
Hypokalaemia	0	0	1 (17%)	1 (17%)	1 (10%)	1 (10%)
ALP increased	1 (25%)	1 (25%)	0	0	1 (10%)	1 (10%)

Abbreviations *CTCAE* Common Toxicity Criteria for Adverse Events, *ALP* alkaline phosphatase

*No grade 5 events occurred in any patient

**Table 5:** Adverse events (by CTCAE version 4.03), regardless of causality, occurring in all cycles of treatment

Dose level	320 mg (*n* = 4)		400 mg (*n* = 6)		All patients (*n* = 10)	
	All grades	Grade ≥ 3	All grades	Grade ≥ 3	All grades	Grade ≥ 3
Diarrhoea	3 (75%)	0	5 (83%)	2 (33%)	8 (80%)	2 (20%)
Rash	3 (75%)	0	4 (67%)	3 (50%)	7 (70%)	3 (30%)
Pruritus	3 (75%)	1 (25%)	1 (17%)	0	4 (40%)	1 (10%)
Neutropenia	0	0	3 (50%)	3 (50%)	3 (30%)	3 (30%)
Fever	0	0	3 (50%)	1 (17%)	3 (30%)	1 (10%)
Febrile neutropenia	0	0	2 (33%)	2 (33%)	2 (20%)	2 (20%)
Infection	1 (25%)	1 (25%)	1 (17%)	0	2 (20%)	1 (10%)
Thromboembolic event	0	0	1 (17%)	1 (17%)	1 (10%)	1 (10%)
Urinary Tract Infection	1 (25%)	1 (25%)	0	0	1 (10%)	1 (10%)
Hypokalaemia	0	0	1 (17%)	1 (17%)	1 (10%)	1 (10%)
Hyperkalaemia	0	0	1 (17%)	1 (17%)	1 (10%)	1 (10%)
ALP increased	1 (25%)	1 (25%)	0	0	1 (10%)	1 (10%)

Abbreviations *CTCAE* Common Toxicity Criteria for Adverse Events, *ALP* alkaline phosphatase

**Fig. 2:**
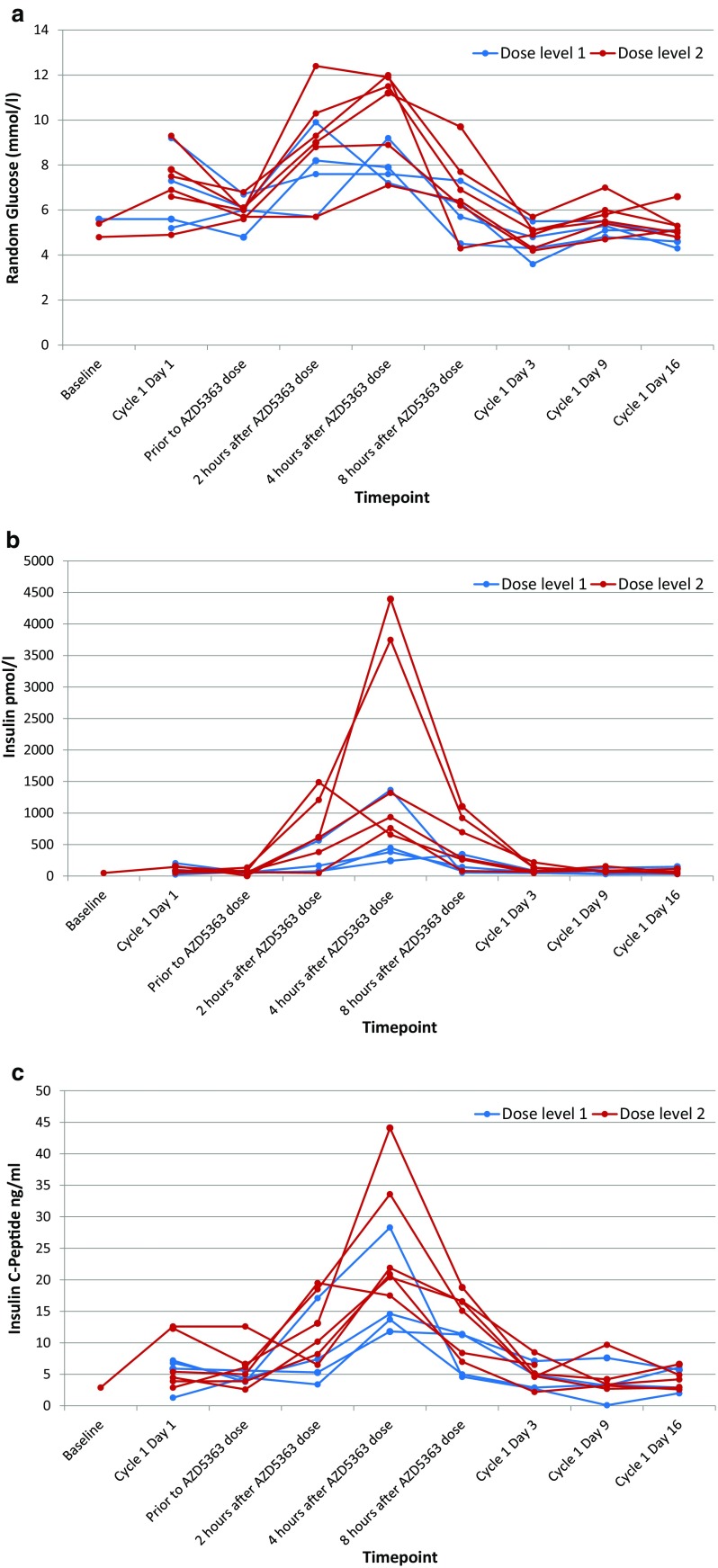
Individual patient data for **a** plasma glucose, **b** insulin and (**c**) C-peptide levels at the indicated time points during cycle 1 of treatment. Samples on day 3, 9 and 16 of the cycle were taken prior to the morning AZD5363 dose

Transient hyperglycaemia is an expected AE for both AZD5363 and corticosteroids. Dexamethasone was used as chemotherapy pre-medication and prednisolone is routinely administered with docetaxel for mCRPC [2]. We saw hyperglycaemia in every patient. The mean random glucose on cycle 1 day 2 (first dose of AZD5363) was 6.0 mmol/L pre-dose and then 8.7 mmol/L at 2 h, 9.5 mmol/L at 4 h, and 6.5 mmol/L at 8 h post AZD5363 dose. Insulin and C-peptide levels also rose and fell in parallel to these glucose changes in all patients. Individual patient data are shown in Fig. 2. No patient experienced symptomatic complications as a result of hyperglycaemia or required intervention to correct a high glucose level (an algorithm, including criteria for metformin administration, was provided within the trial protocol).

### Pharmacokinetics

Pharmacokinetic analyses indicated that AZD5363 exposure for patients receiving concomitant DP chemotherapy was in keeping with that found in patients receiving mono-therapy in the AZD5363 development programme.

### Treatment efficacy

**Fig. 3:**
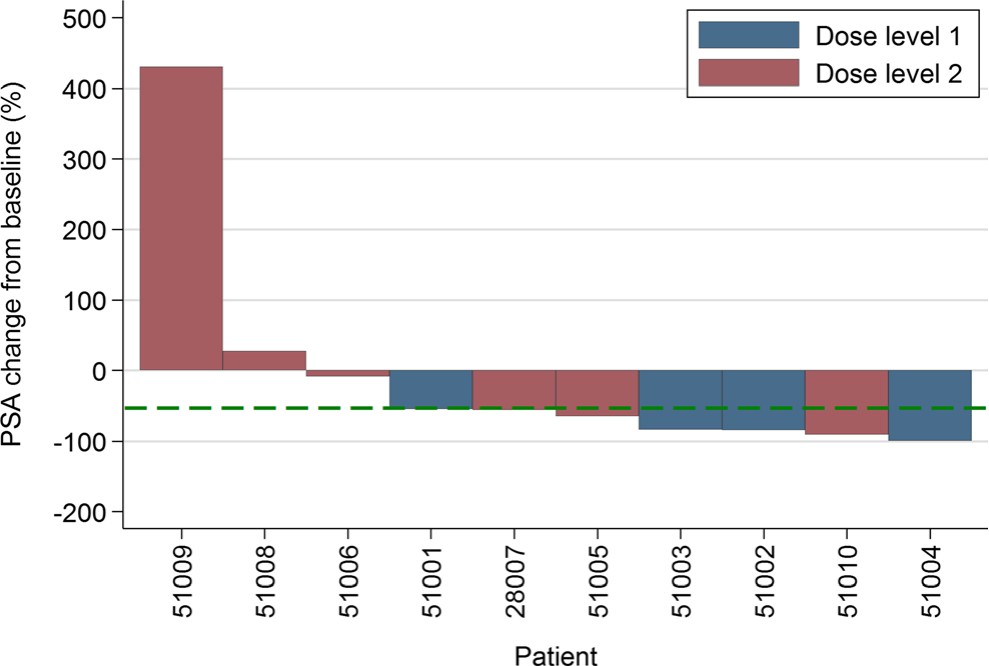
Percentage change from baseline of individual patient prostate specific antigen (PSA) level after 12 weeks of therapy

PSA reduction from baseline level to <50% (PSA_50_) at 12 weeks of treatment was seen in 7 (70%) of patients (Fig. 3).

## Discussion

We undertook a phase I, open-label, combination dose escalation trial to determine an appropriate dose for AZD5363 to be used in combination with conventional doses of DP chemotherapy for use in mCRPC. The resulting RP2D for AZD5363 in this combination is 320 mg, BID, PO, given according to a 4 days on and 3 days off schedule and commencing from cycle 1, day 2 based on pre-defined criteria for dose limiting toxicity. In addition we found that the higher AZD5363 dose level of 400 mg BID resulted in 4 of 6 patients discontinuing AZD5363 within the first 2 cycles of treatment despite being able to tolerate further DP cycles. This supports the selection of the 320 mg BID dose level for this combination, which by comparison was tolerable at a level broadly consistent with the use of DP alone in this setting over multiple cycles [2, 3].

The most common AEs which were considered to be associated with the addition of AZD5363 to chemotherapy were diarrhoea, rash, neutropenia and hyperglycaemia in keeping with the experience to date of single agent AZD5363 administration [18, 19]. Diarrhoea and rash responded in most patients to management with loperamide and systemic anti-histamines with most patients able to continue therapy in the face of these AEs. We did not see evidence of increased nausea, at least above that experienced with DP, which has been an AE in single agent studies.

Hyperglycaemia occurred in all patients who received AZD5363 but was transient and self-limiting within 8 h of the first AZD5363 dose. We had chosen a 4 days on 3 days off schedule for dosing of AZD5363. This was one approach under evaluation within single agent AZD5363 trials at the point that our trial was designed. Our choice of this schedule was driven, in part, to allow us to separate temporally the administration of AZD5363 from the relatively high doses of dexamethasone pre-medication administered prior to docetaxel in an attempt to reduce the potential for interaction to drive hyperglycaemia. This strategy appears to have been successful and despite the concurrent use of prednisolone at comparatively lower doses. Of note, our study excluded patients with type I or II diabetes mellitus requiring either insulin or oral hypoglycaemics for routine management which should be borne in mind in the subsequent development of this approach.

This study was not designed to evaluate the efficacy of this combination at this stage of development. The PSA_50_ response rate of 70% of patients, and the fact that we administered a median of 8.5 cycles of chemotherapy is broadly consistent with prior data from large randomised trials of DP in this setting [2, 8]. However, we are unable to determine on this current experience what additional benefit AZD5363 might add to DP alone in this setting which will form the basis of future development of this combination. On the basis of our results, recruitment is currently ongoing to a placebo-controlled randomised phase II trial in mCRPC to evaluate the impact on progression free survival of the addition of AZD5363 to DP compared to DP alone. Collection of tissue samples, and blood sampling for circulating biomarkers will allow us to undertake exploratory analyses within this latter trial for patient subsets that might benefit based on activation of the PI3K/AKT signalling pathway.

Our determination of a proposed RP2D at 320 mg BID on a 4 day on/3 day off schedule for combination with DP was based on the treatment of 4 patients at this dose level, of which 3 were fully evaluable. Within a standalone trial this would have required a dose level expansion cohort to confirm tolerability. Our approach instead has been to confirm tolerability through direct progression to a placebo controlled, randomised, phase II component of the trial which is currently recruiting. Patients are randomised (1:1) to DP/AZD5363 or DP/placebo. Following recruitment of the first 20 randomised patients, a prospectively planned review by a fully independent Data Monitoring and Ethics Committee (DMEC) has considered toxicity and tolerability data to which the DMEC were unblinded. On the basis of this review, the committee have recommended ongoing recruitment. This approach to undertaking a dose level expansion cohort was chosen to provide efficiencies in moving through the development of this treatment approach such that these patients will also contribute to the subsequent phase II efficacy assessment based on a primary endpoint of progression free survival.

AKT inhibition utilising AZD5363 is also being developed in other combination approaches. Of note, RE-AKT is an ongoing multicentre, randomised, phase I/II trial in mCRPC, evaluating a combination of AZD5363 with the androgen receptor antagonist enzalutamide (https://clinicaltrials.gov/ct2/show/NCT02525068) [22]. In addition, analogous data for a taxane combination has been reported in breast cancer for AZD5363 plus paclitaxel, with the same 4 day on/3 day off AZD5363 schedule [23]. Toxicities were broadly similar, as was the RP2D, although at a dose level higher, at 400 mg BID, giving further reassurance that our RP2D of 320 mg BID is likely to prove tolerable.

In conclusion, AZD5363 at a dose of 320 mg BID on a 4 day on/3 day off schedule is recommended for further evaluation in combination with full dose DP for mCRPC.

## Electronic supplementary material

ESM 1 (DOCX 20 kb)
