# Supplementary material for: ProCAID: a phase I clinical trial to combine the AKT inhibitor AZD5363 with docetaxel and prednisolone chemotherapy for metastatic castration resistant prostate cancer
Source: Invest New Drugs. 2017 Feb 1;35(5):599–607. doi: 10.1007/s10637-017-0433-4 (PMC5613074; doi:10.1007/s10637-017-0433-4)
Supplement: Supplementary file 1 — (DOCX 20 kb) [file 10637_2017_433_MOESM1_ESM.docx]

**Supplementary Appendix: Eligibility criteria for patient entry**

The protocol for the ProCAID phase I trial listed the following as inclusion and exclusion criteria for patient entry.

**Inclusion criteria:**

Patients will be required to meet the following criteria in full:

1. Histologically or cytologically proven mCRPC with documented metastases (measurable or evaluable disease is acceptable) now eligible for treatment with docetaxel chemotherapy
2. Disease progression since the last change in therapy defined by one or more of the following according to the Prostate Cancer Working Group (PCWG2) criteria (Scher et al. 2008 J Clin Oncol. 26; 1148):
   1. PSA progression as defined by the prostate cancer working group 2 (PCWG2) criteria (Scher et al. 2008 J Clin Oncol. 26; 1148). This must be based on a series of at least 3 readings at least 7 days apart demonstrating rising PSA. The 3rd reading must be ≥ 2ng/ml. In the event where an intermediate reading is lower than a previous reading, then the patient will still be eligible (i.e. the 3 readings do not need to be consecutive). The first of the three readings must have been obtained after commencing the previous systemic therapy, or, in the case of androgen receptor antagonists, after discontinuing.
   2. Radiographic progression of nodal or visceral metastases as defined by RECIST version 1.1
   3. iii. The appearance of two or more new bony metastases
3. Serum testosterone <1.7 nmol/L (ongoing LHRH analogue or antagonist therapy is permitted to maintain a castrate state)
4. Discontinuation of prior therapies for prostate cancer ≥ 4 weeks prior to commencing study treatment (with the exception of an LHRH agonist or antagonist where required for ongoing testosterone suppression)
5. No anti-androgen withdrawal response. Consistent with PCWG2 guidelines, investigators should evaluate patients to exclude withdrawal response for 6 weeks after stopping anti-androgen therapy. Investigators need not wait to assess for withdrawal response in patients who did not respond, or who showed a PSA decline for ≤ 3 months, after an anti-androgen was administered as a second-line or later intervention
6. ECOG performance status 0 or 1
7. Hb ≥ 9g/dL; platelets ≥ 100 x 10^9^/L; neutrophils ≥ 1.5 x10^9^/L
8. Bilirubin ≤ ULN ; ALT and AST ≤ 1.5 x ULN
9. Sodium and potassium within the normal range for the treating institution
10. Able to swallow oral study drugs (without crushing/opening in the case of AZD5363)
11. Life expectancy > 3 months
12. Aged 18 years or over
13. Provision of written informed consent

**Exclusion Criteria:**

Patients with any of the following are ineligible for this study:

1. Previous treatment with cytotoxic chemotherapy (patients may have received previous or ongoing bisphosphonates or denosumab). There are no restrictions on prior use of second generation hormonal therapies e.g. abiraterone, enzalutamide
2. Prior malignancy with an estimated ≥ 30% chance of relapse within 2 years
3. Previously identified brain metastases, or spinal cord compression unless treated with full functional recovery
4. Prior radiotherapy to > 30% of bone marrow
5. Administration of an investigational agent within 30 days of first dose of study medication
6. Type I or II diabetes mellitus requiring either insulin or oral hypoglycaemics for routine management. Patients with type II diabetes mellitus that is well controlled by dietary measures alone are eligible to participate. Patients found to have a fasting glucose ≥7 mmol/L (≥126 mg/dL) or glycosylated haemoglobin >8% (64 mmol/mol) at screening should be assessed for appropriate management according to local policy.
7. Malabsorption syndrome, previous gastrointestinal surgery, or other gastrointestinal condition that may affect drug absorption
8. Coronary artery bypass graft, angioplasty, vascular stent, myocardial infarction, angina pectoris or congestive heart failure (NYHA ≥ grade 2) within the last 6 months
9. Abnormal echocardiogram (LVEF <LLN)
10. Uncontrolled hypotension (systolic blood pressure <90 mmHg and/or diastolic blood pressure <50 mmHg)
11. QTc interval of >480 msec at two or more time points within a 24 hour period
12. Proteinuria (either 3+ on dipstick analysis or >500 mg/24 hours) or creatinine >1.5 x ULN concurrent with creatinine clearance <50 mL/min (assessed as per local practice e.g. by Cockcroft and Gault estimation)
13. Exposure to potent inhibitors or inducers of CYP3A4 or CYP2D6 or substrates of CYP3A4 within 2 weeks before the first dose of study treatment (3 weeks for St John’s Wort)
14. Unresolved toxicity ≥ grade 2 (except alopecia) from previous cancer therapy
15. Patients with a partner of child-bearing potential who are not using a highly effective method of contraception, who are unwilling to use condoms during the study and for 30 days after the last dose of study drug
16. Known hypersensitivity to AZD5363, its excipients, or drugs in its class
17. Previous exposure to agents with the following mechanisms of action:

- inhibition of AKT (e.g., MK2206, GDC0068, GSK2110183, GSK2141795) any inhibitor with PI3K pharmacology (e.g., GDC0941, XL147, BKM120, PX866, BYL719, AMG319, GDC0032, INK1117, INK119)
- any compound with mixed PI3K and mammalian target of rapamycin (mTOR) kinase pharmacology (e.g., BEZ235, GDC0980, PF04691502, PF05212384, GSK2126458, XL765)
- or any mTOR kinase inhibitor (e.g., AZD8055, AZD2014, OSI027, INK128)
